# Meta‐analysis of Gaussian individual patient data: Two‐stage or not two‐stage?

**DOI:** 10.1002/sim.7589

**Published:** 2018-01-18

**Authors:** Tim P. Morris, David J. Fisher, Michael G. Kenward, James R. Carpenter

**Affiliations:** ^1^ London Hub for Trials Methodology Research MRC Clinical Trials Unit at UCL London UK; ^2^ Ashkirk UK; ^3^ Department of Medical Statistics London School of Hygiene and Tropical Medicine London UK

**Keywords:** individual‐patient data, meta‐analysis, one‐stage, two‐stage

## Abstract

Quantitative evidence synthesis through meta‐analysis is central to evidence‐based medicine. For well‐documented reasons, the meta‐analysis of individual patient data is held in higher regard than aggregate data. With access to individual patient data, the analysis is not restricted to a “two‐stage” approach (combining estimates and standard errors) but can estimate parameters of interest by fitting a single model to all of the data, a so‐called “one‐stage” analysis. There has been debate about the merits of one‐ and two‐stage analysis. Arguments for one‐stage analysis have typically noted that a wider range of models can be fitted and overall estimates may be more precise. The two‐stage side has emphasised that the models that can be fitted in two stages are sufficient to answer the relevant questions, with less scope for mistakes because there are fewer modelling choices to be made in the two‐stage approach. For Gaussian data, we consider the statistical arguments for flexibility and precision in small‐sample settings. Regarding flexibility, several of the models that can be fitted only in one stage may not be of serious interest to most meta‐analysis practitioners. Regarding precision, we consider fixed‐ and random‐effects meta‐analysis and see that, for a model making certain assumptions, the number of stages used to fit this model is irrelevant; the precision will be approximately equal. Meta‐analysts should choose modelling assumptions carefully. Sometimes relevant models can only be fitted in one stage. Otherwise, meta‐analysts are free to use whichever procedure is most convenient to fit the identified model.

## 1 INTRODUCTION

Quantitative synthesis of evidence from multiple studies through meta‐analysis is a cornerstone of medical, psychological, and social research. Historically, parameter estimation in meta‐analysis has involved two stages, taking (published) estimates of effect and standard errors from each study (first stage), then combining them (second stage), assigning weights to each study, with weights most often based on the inverse variance.

Over the last 2 decades, the bar for high‐quality systematic reviews has been raised, particularly by reviewers who have collected participant‐level data for included studies and analysed these datasets to obtain their own trial‐level estimates in place of published estimates. The merits of using individual participant data (IPD) are well documented^1^, ^2^, ^3^, ^4^: Data can be checked by the reviewer; consistent inclusion and exclusion criteria can be applied; participants can be included who were not in the original reports; longer follow‐up may be included than at the time of the primary study report; analyses can be executed identically for each study, meaning that it is more reasonable to combine estimates; and there is greater scope to investigate interactions. Collecting and checking IPD is a time‐consuming, diplomatic, and expensive exercise, so when IPD has been obtained, the advantages should be fully leveraged.

When IPD have been obtained, the analysis is not restricted to combining estimates and standard errors from published data. Instead, the whole analysis can be done in one stage. The merits of “one‐stage” compared to “two‐stage” approaches have been debated.^5^, ^6^, ^7^, ^8^ Those who argue for one‐stage methods tend to be statistically trained, while those who argue for two‐stage methods are typically meta‐analysts whose primary training is not statistical. (Executing a thorough systematic review and IPD meta‐analysis involves many non‐statistical skills.) There seems to be support for both perspectives.

The views of a one‐stage exponent are exemplified by an anonymous reviewer of one of the authors' (DJF) work on a Stata package to perform two‐stage meta‐analysis of IPD.^3^ The reviewer was ambivalent. Despite approving the package, he or she was convinced that two‐stage methods have little to offer, writing

The debate is ongoing but the statistical merits of one‐stage IPD far outweigh the benefits of a two‐stage approach … 

… the merit of the one‐stage is doing an analysis that is definitely more accurate …

… a simulation study is needed to quantify how much better one‐stage is in certain scenarios …

I do not think it [two‐stage meta‐analysis] should be recommended for use when a one‐stage approach is possible …

These opinions were apparently informed by Mathew and Nordström's paper,^6^ which shows that a two‐stage procedure can be at best as asymptotically efficient as a one‐stage procedure. This is in contrast to the view of Burke et al who argue that “differences arise because of different modelling assumptions, rather than the choice of one‐stage or two‐stage itself.”^9^

The two‐stage perspective tends to be that the models and their assumptions are transparent and of scientific value and are sufficient to address relevant substantive questions. There is arguably less transparency in the reporting of models used with one‐stage analysis, as evidenced by two recent reviews.^10^, ^11^ Simmonds et al found that “One‐stage methods were in general more poorly described, perhaps because of the greater complexity involved in describing properties of regression models.”^10^ The main choices for two‐stage analysis are around whether to model treatment effects as fixed or random and (a closely related choice) how much weight studies should contribute to the overall estimate. The apparent simplicity of two‐stage analysis, combined with the longer history of two‐stage estimation in the context of meta‐analysis, may be why descriptions tend to be better.

The aim of this paper is to consider the statistical arguments for one‐ and two‐stage analysis in the context of Gaussian outcome data, and particularly to identify differences in precision for models that can be fitted in either way. This is done in the finite‐sample setting; the reality we all work in. That is, our results allow explicitly for small numbers of patients‐per‐study and the implied uncertainty about study‐specific estimates.

To aid clarity, we focus here on the simple setting where the IPD include a quantitative outcome, an indicator of treatment assignment, a participant identifier, and a study identifier. It is assumed that the focus of the meta‐analysis is on the treatment effect and that no covariates are present. This is an inadequate summary of a meta‐analysis,^12^ but this is often the primary focus and permits a clear discussion of the arguments for and against one‐ and two‐stage approaches.

As evidenced by the above quotes, two‐stage procedures can receive a bad press from statisticians, and we seek to establish the extent to which such comments are justified, not asymptotically, but in practice.

## 2 MODELS FOR META‐ANALYSIS

Even for the relatively simple structure of datasets considered here, there are several possible models for performing a meta‐analysis. Borrowing heavily from Senn,^13^ Table 1 lists seven that might be of interest. We define our notation below before discussing the models in Table 1.

**Table 1:** Models for individual patient data meta‐analysis. Model II is the “fixed‐effects meta‐analysis” and model V is the “random‐effects meta‐analysis”

	Model	$\sigma_{i}^{2}$	one‐stage?	Example one‐stage Stata code
			two‐stage?	Example two‐stage Stata code
I	rl*y*_*ij*_=*α*+*β* *x*_*ij*_+*ε*_*ij*_	Common	Yes	regress y x
			No	…
II	rl*y*_*ij*_=(*α*+*a*_*i*_)+*β* *x*_*ij*_+*ε*_*ij*_	Study‐specific	Yes	mixed y x i.study, residuals(,by(study)) reml
			Yes	ipdmetan, study(study): regress y x
III	*y*_*ij*_=*α*_*i*_+*β* *x*_*ij*_+*ε*_*ij*_	Common	Yes	mixed y x || study: , reml
	*α*_*i*_=*α*+*a*_*i*_where*a*_*i*_∼*N*(0,*λ* ^2^)		No	…
IV	*y*_*ij*_=(*α*+*a*_*i*_)+(*β*+*b*_*i*_)*x*_*ij*_+*ε*_*ij*_	Common	Yes	regress y x#study
			No	…
V	*y*_*ij*_=(*α*+*a*_*i*_)+*β*_*i*_ *x*_*ij*_+*ε*_*ij*_	Study‐specific	Yes	mixed y x i.study || study: x, nocons residuals(,by(study)) reml df(kr)
	*β*_*i*_=*β*+*b*_*i*_where*b*_*i*_∼*N*(0,*τ* ^2^)		Yes	^*b*^ ipdmetan, study(study) re(kr): regress y x
VI	*y*_*ij*_=*α*_*i*_+(*β*+*b*_*i*_)*x*_*ij*_+*ε*_*ij*_	Study‐specific	Yes	mixed y x#study || study: , residuals(,by(study)) reml df(kr)
	*α*_*i*_=*α*+*a*_*i*_where*a*_*i*_∼*N*(0,*λ* ^2^).		Yes^*a*^	^*b*^ ipdmetan, study(study) re poolvar(_cons): regress y x
VII	*y*_*ij*_=*α*_*i*_+*β*_*i*_ *x*_*ij*_+*ε*_*ij*_
	*α*_*i*_=*α*+*a*_*i*_and	Study‐specific	Yes	mixed y x || study: x, residuals(,by(study)) reml
	*β*_*i*_=*β*+*b*_*i*_where		Yes	^*b*^ mvmeta_make regress y x, by(study) saving(data) replace names(b V)
	(*a*_*i*_,*b*_*i*_)∼BVN $\left(\left[\begin{array}{c} 0\\ 0 \end{array}\right],\right.$ $\left.\left[\begin{array}{cc} \lambda & \rho \\ \rho & \tau \end{array}\right]\right)$			then ^*b*^ mvmeta b V, reml

^a^While the model can technically be fitted in a two‐stage way, it only obtains an estimate of *α*, not *β*so is practically useless for meta‐analysis of treatment effects.

^b^Note that ipdmetan and mvmeta are user‐written Stata packages, which can be installed in Stata using “. ssc install command_name.”

### 2.1 Notation

Let *y* denote outcome and *x* denote treatment assignment^14^ (coded −0.5/+0.5). Let *i*=1,…,*I* index studies and *j*=1,…,*n*_*i*_ index patients within a study, so that *y*_*ij*_ is the outcome for the *j*th patient in the *i*th study. Let *α* be the intercept term and *β* be the treatment effect, the parameter of central interest. The interpretation of *α* and *β* will depend on which model is used; for example, in model I, *α* represents mean outcome (because treatment is coded −0.5/+0.5 and it is assumed that randomisation is 1:1).

There are 3 possibilities for modelling the main effect (intercept) of study:

*α*_*i*_=*α*,∀*i*: a single constant; studies share an intercept
*α*_*i*_=*α*+*a*_*i*_: fixed‐study intercept
*α*_*i*_=*α*+*a*_*i*_ where*a*_*i*_∼*N*(0,*λ*^2^): random‐study intercept

Similarly, for the treatment effect,

1 *β*_*i*_=*β*,∀*i*
2 common treatment effect (no interaction)
3 *β*_*i*_=*β*+*b*_*i*_
4 fixed treatment effects which are different for each study
5 (fixed treatment‐by‐study interaction terms)
6 *β*_*i*_=*β*+*b*_*i*_ where*b*_*i*_∼*N*(0,*τ* ^2^)
7 random treatment effect
8 (random treatment‐by‐study interaction)

Let $\varepsilon_{ij}∼N(0,\sigma_{i}^{2})$. With a one‐stage analysis, $\sigma_{i}^{2}$ can be allowed to vary by study or restricted such that $\sigma_{i}^{2}=\sigma^{2}\forall i$ or some combination of these. For example, a fixed‐effects model based on ordinary least squares (OLS) implies *α*_*i*_=*α*+*a*_*i*_ (with *a*_*i*_ representing fixed differences in intercepts for *i*=1,…,), *β*_*i*_=*β*, and $\sigma_{i}^{2}=\sigma^{2}$. Two‐stage analysis by default allows $\sigma_{i}^{2}$ to vary by *i*(we have referred to this as *default* because, at the time of writing, we believe all software packages which perform stage two do so under this assumption). However, the assumption that $\sigma_{i}^{2}=\sigma^{2}$ can also be invoked with a two‐stage analysis (see Olkin and Sampson^5^. To do this, relative study weights are proportional to a function of *n*_*i*_ rather than of ${\hat{\sigma }}_{i}^{2}$. Weighting in this way exactly recovers the one‐stage OLS estimate.^15^ We return to this point later.

We distinguish here between two types of variance we will refer to, particularly for understanding the workings provided in the appendices. We denote the true variance of the estimator of *β* by $\mathrm{Var}(\hat{\beta })$. In the frequentist sense, this is the long‐run variance of $\hat{\beta }$ under repeated sampling. Second, the estimated variance is denoted by $\hat{\mathrm{Var}}(\hat{\beta })$. This is what is estimated in a specific realisation of a meta‐analysis. A good variance estimator should have expectation $\mathrm{Var}(\hat{\beta })$.

### 2.2 Flexibility and estimands

Note the column of Table 1 stating whether the model can be fitted in one stage (top) or two (bottom). Of the models considered, (I), (III), and (IV) cannot be fitted in two stages. Further, although model (VI) can be fitted in two stages, it does not provide an estimate of *β*. This restriction is what is meant when one‐stage approaches are promoted for their flexibility.

Note that, provided that the available evidence is representative and the model specific assumptions hold, any of the models listed in Table 1 could provide an unbiased estimate of the treatment effect and its variance. The practical settings for which this is true may be rather limited. For example, if each study used simple randomisation and the same allocation ratio (not necessarily 1:1), then $\hat{\beta }$ would be unbiased for all the models in Table 1. The interesting point is that this depends only on the design of the included studies and not on the true data‐generating model. We refer interested readers to Parzen et al^16^ or Kahan and Morris.^17^

## 3 A COMPARISON OF ONE‐ AND TWO‐STAGE APPROACHES FOR LINEAR MODELS

### 3.1 Fixed‐effects model (model II)

We wish to compare the variance of one‐ and two‐stage fixed‐effect meta‐analysis. There are 2 approaches to this.

1. Compare the variance of “default” or most natural one‐ and two‐stage fixed‐effect estimators.
2. Compare the variance of one‐ and two‐stage estimators when the models and associated assumptions are the same.

The former is an apples‐vs‐oranges comparison. It is interesting because one‐stage fixed‐effect estimators are most naturally estimated by OLS (this is the default in all major software), which assumes a common variance across studies (previously described as “scarcely credible” in the context of meta‐analysis).^15^ Meanwhile the two‐stage inverse‐variance estimator most naturally assumes heterogeneity of variances across studies (again this is the default in software). Assumptions about within‐study variances impact on the relative weights of studies and thus on the overall estimate of *β* and its variance. Results may differ between one‐ and two‐stage analysis due to subtle differences in modelling assumptions, such as this one. It is important not to attribute this to the number of stages in computation. Therefore, the comparison of defaults is very much a comparison of the modelling assumptions, rather than of the procedure by which the models are estimated (ie, in one or two stages).

The most natural one‐stage analysis is to fit a model based on OLS, where the theory is well understood. This assumes $\sigma_{i}^{2}$ is common across studies. The theory is less well understood when $\sigma_{i}^{2}$ are allowed to be study specific, and the model is estimated based on variance‐weighted least squares. The fact that the mixed command is needed to fit this model demonstrates that it is not a natural one‐stage model to fit. We derive the one‐stage estimator of *β* and variance estimator allowing study‐specific $\sigma_{i}^{2}$ in Appendix A.1. These turn out to be the same as the two‐stage inverse‐variance estimator and the usual asymptotic inverse‐variance formula:

(1) $$\hat{\mathrm{Var}}(\hat{\beta })=\sum_{i=1}^{I}\frac{4{\hat{\sigma }}_{i}^{2}}{n_{i}}.$$

For details, see Appendix A.1.

Thus, when we allow study‐specific variances, computing our estimates in one or two stages would lead to identical point estimates and variance estimates. (In fact, the variance estimators are biased downwards in finite samples because they assume that the ${\hat{\sigma }}_{i}^{2}$ are known rather than estimated, as is assumed in Mathew and Nordström.^6^ This result is related to the result of Olkin and Sampson,^5^ when both models impose a shared $\sigma_{i}^{2}$, and to Lunn et al,^18^ who present a two‐stage strategy for fitting a full Bayesian model.

We focus on comparisons of variance here and, to do so, needed to derive some new theoretical results. Appendix A works through the mathematics, while the rationale and results are given here. The overall aim is to calculate the expected value of the variance for the OLS and inverse‐variance estimators. This involved:

1. Calculation of the expected value of the asymptotic variance formula (1) for the inverse‐variance estimator (noting again that this variance estimator is biased downwards in small samples and so produces misleading conclusions) (Appendix A.2).
2. Derivation of a new variance formula which explicitly acknowledges that the ${\hat{\sigma }}_{i}^{2}$ are estimates and so is unbiased with practical sample sizes (Appendix A.3). This tends to the standard formula (1) as all *n*_*i*_→*∞* but is less biased when any *n*_*i*_ is small. This is
  (2) $$\mathrm{Var}(\hat{\beta })≃\frac{1}{\hat{W}}+\sum_{i=1}^{I}\frac{2\sigma_{i}^{4}}{n_{i}-1}{\left(\frac{{\hat{\beta }}_{i}-\hat{\beta }}{\hat{W}}\right)}^{2},$$
  where $1/\hat{W}$ is the standard inverse‐variance formula (1).
3. Calculation of the expectation of the new small‐sample variance formula (2) (Appendix A.4).

The expected value of the asymptotic and small‐sample variance formulas are then compared with the version that assumes common ${\hat{\sigma }}_{i}^{2}$.

**Figure 1:**
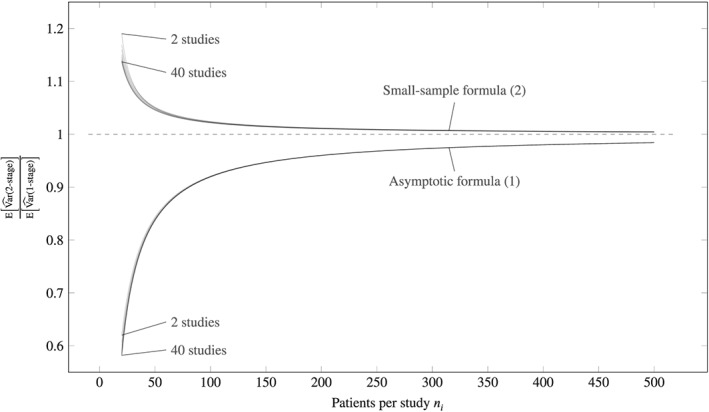
Ratio of two‐ to one‐stage expected value of variances, using the asymptotic variance formula (1) (lines below 1) and the small sample formula (2) (lines above 1). The plot shows 39 translucent lines as the number of studies increases from i=2,…,40. The lines for smaller i are higher for both panels, but differences are negligible

Results are shown in Figure 1, showing very little effect of the number of studies *I* (the 40 translucent curves are almost on top of each other) but some effect of the number of patients per study *n*_*i*_. However, this effect becomes small with more than about 25 patients per study. This is shown for scenarios where all studies contain the same number of participants but could equally be done with specific incidences of unbalanced data.

This demonstrates that assuming $\sigma_{i}^{2}$ is the same across studies reduces variance of $\hat{\beta }$ when *n*_*i*_ is small. This being the assumption underlying OLS, it is typically associated with one‐stage analysis. Two‐stage fixed‐effects meta‐analysis typically allows $\sigma_{i}^{2}$ to be study specific. These are merely defaults, and it is not necessary for either procedure to use the default assumption.

### 3.2 Random‐effects model (model V)

Here, we consider model V of Table 1, often designated as “random‐effects” meta‐analysis, because the *I* treatment effects are assumed to follow a probability distribution. There is a bewildering range of competing two‐stage estimators for this model.^3^

We will consider the restricted maximum likelihood estimator. It is attractive because it is more efficient than moment‐based estimators such as DerSimonian and Laird's method but can provide consistent estimates of variance parameters. For finite‐sample inference with restricted maximum likelihood (REML) estimation, Kenward and Roger's approximate correction to the covariance matrix and degrees of freedom is the best available option.^19^

It is possible to fit the random‐effects meta‐analysis REML realisation of the Kenward–Roger correction in one stage via mixed in Stata or proc mixed in sas. The covariance and degrees of freedom for two‐stage meta‐analysis of a single parameter has not previously been derived; we provide the derivation in Appendix B.

The one‐ and two‐stage versions of these models were compared in a simulation study. The simulation design follows. This is structured as aims, data‐generating mechanism, methods, estimand, and performance measures.

- **Aims**
  The aim of the simulation study is to compare one‐ and two‐stage procedures in terms of (1) precision of meta‐analysis with random treatment effects and fixed‐study intercepts and (2) coverage of 95% confidence intervals. Both are with respect to *β*, the overall average treatment effect.
- **Data‐generating mechanism**
  Individual participant data were simulated for independently 2 up to 40 studies from model V of Table 1, that is, studies had fixed intercepts and random treatment effects (drawn from a Gaussian distribution). One thousand simulated datasets were produced using Stata 14's default random‐number generator (64‐bit Mersenne twister). For the data‐generating mechanism we used, parameter values were set to *α*=*a*_*i*_=0∀*i*,*β*=0 with $E[{\hat{\tau }}^{2}]=1$, and $E[{\hat{\sigma }}^{2}]=1$ with $\mathrm{Var}({\hat{\sigma }}^{2})=50/\chi_{50}^{2}$(where $\chi_{50}^{2}$ denotes a random draw from a *χ* ^2^ distribution on 50 df). Study sizes were unbalanced. Following Rücker et al,^20^ based on Galandi et al,^21^ study sizes *n*_*i*_ were drawn from a log‐normal distribution with $E(logn_{i})=3.798$ and $\text{SD}(logn_{i})=1.104$, with *n*_*i*_ rounded to the nearest integer or to 20 for any drawn values <20.
- **Methods**
  One‐stage analysis was done in sas 9.3 using proc mixed, while two‐stage analysis^3^ used ipdmetan in Stata 14, based on the same simulated datasets. The choice of sas for one‐stage models was for computational speed: Both packages return the same results, but sas currently gets there faster. The only difference in the methods is that the two‐stage variance estimates were based on expected information and the one‐stage on observed.
- **Estimand**
  The estimand of interest is the meta‐analytic estimate of average overall treatment effect *β*. As earlier mentioned, we acknowledge that this is not an adequate summary of a meta‐analysis but is usually the parameter of central interest.
- **Performance measures**
  The key performance measure is precision: the inverse of the empirical variance of $\hat{\beta }$.^22^ This is estimated for two‐stage relative to one‐stage (see White^22^ and presented as “% gain”. The empirical variance is based only on the REML point estimate and so does not depend on the Kenward–Roger adjustment. To assess the new variance and df adjustments, we also compare the coverage of nominal 95% confidence intervals.

The Stata files required to generate data and run one‐ and two‐stage analyses are included as a supplement to this article. Also included is the simulation analysis of the results (though the code to produce figures is not).^22^

**Figure 2:**
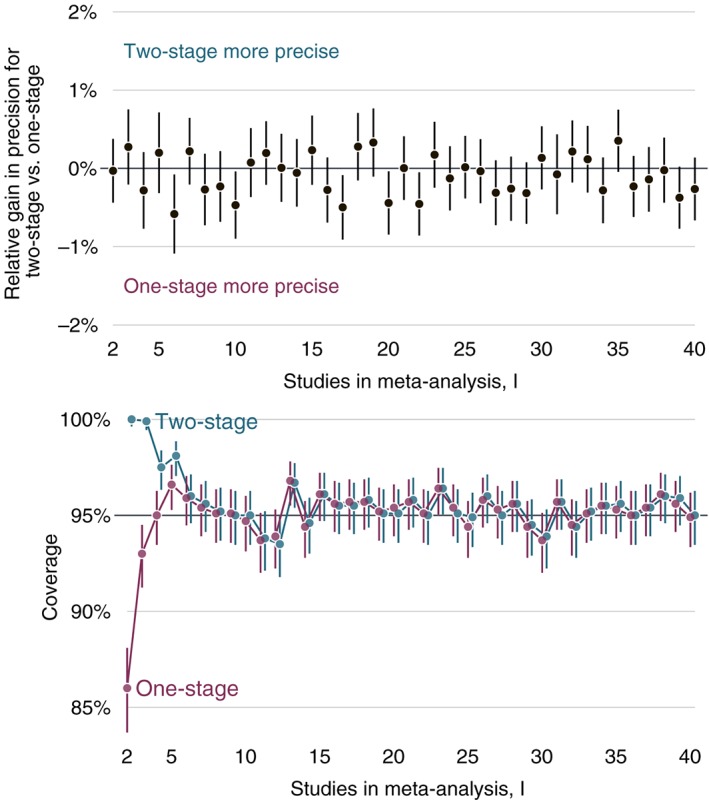
Simulation study results from 1000 repetitions plotted for 2 up to 40 studies in a meta‐analysis. Upper panel: relative % increase in precision for two‐ vs one‐stage restricted maximum likelihood estimation. Lower panel: coverage of Kenward–Roger confidence intervals

Figure 2 plots results for relative precision and coverage. All results are accompanied by 95% Monte Carlo confidence intervals to describe simulation uncertainty.^22^

The upper panel displays relative precision, showing that the two‐stage REML estimator has precision extremely similar to its one‐stage counterpart under this data‐generating mechanism. Although we are not able to obtain an analytic result to support whether this is a general or specific result, it appears to be in line with the equivalence of one‐ and two‐stage for fixed‐effects meta‐analysis when the same models are fitted.

Coverage results (the lower panel of Figure 2) indicate that both methods tend to have good coverage, except when the number of studies is below 5. Inspection of the simulation results shows that variance estimates are accurate, on average very close to the true (empirical) variance. The conservative intervals appear to be due to the df estimated by two‐stage Kenward–Roger approach being conservative (two‐stage) and anti‐conservative (one‐stage). For both procedures, the variance formulas closely matched the empirical variance of the REML estimate of β. The issue with confidence interval coverage is then due to the df used. A comparison of the one‐ and two‐stage df across simulation runs showed that the one‐stage df were slightly higher than the two‐stage counterpart. This is apparently due to the approximations involved in the Taylor expansions, which begin to fail in different directions at very small study sample sizes.

## 4 ILLUSTRATION OF METHODS FOR INDANA META‐ANALYSIS

The Indana data includes IPD from 10 trials of antihypertensive drugs in patients at high risk of cardiovascular disease.^23^, ^24^ Several outcome variables were collected, such as death, stroke, systolic blood pressure, diastolic blood pressure, and cholesterol. We focus here on systolic blood pressure (SBP), which was recorded annually at years 1 to 5 in seven of the 10 studies and never in the remaining three. As previously, we consider a simple analysis involving a linear regression of SBP on randomised group. Analysis was restricted to participants with observed values of SBP at 1 year.

**Figure 3:**
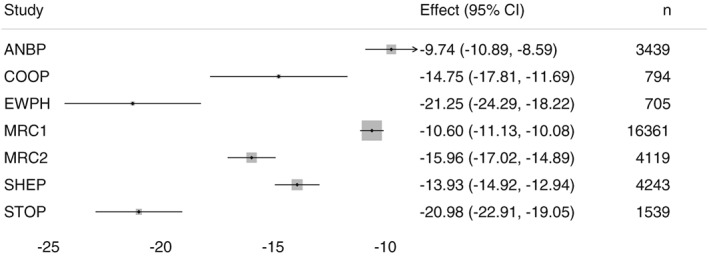
Forest plot of mean difference in systolic blood pressure at 1 year by randomised arm, for 7 trials in the indana data. The size of squares is based on fixed‐effect weights with study‐specific $\sigma_{i}^{2}$. CI, confidence interval

The situation is shown in Figure 3, a forest plot of the within‐study estimates of the mean difference in SBP at 1 year. The trials are highly heterogeneous in many ways, and this is apparent in the estimated treatment effects. A fixed‐effect meta‐analysis will thus produce a confidence interval that is too narrow and relates to what is arguably an ill‐defined target parameter, as the assumption of a constant treatment effect is clearly not supported. Meanwhile, the smaller trials seem to have larger effects, so that the random‐effects point estimate may be too large. Our purpose here is not to produce the most appropriate summary of the overall effect in terms of the point estimate and confidence interval; instead, we aim to demonstrate the impact of different modelling assumptions and of the number of stages used to fit a given model, on these quantities.

We consider three different modelling assumptions, matching the models considered in Section 2. First, a fixed‐effect meta‐analysis where $\sigma_{i}^{2}$ is assumed to be equal in all studies; second, a fixed‐effect meta‐analysis under the assumption that $\sigma_{i}^{2}$ are different across studies; and third, a random‐effects meta‐analysis also allowing $\sigma_{i}^{2}$ to vary across studies. Table 2 gives the results of applying one‐ and two‐stage procedures for parameter estimation. Results are intentionally given to 4 decimal places. We see clear differences between different modelling assumptions, the largest difference being between the fixed‐ and random‐effects models. However, the different procedures produce almost identical estimates of *β* and very similar confidence intervals.

**Table 2:** Results from meta‐analysis of INDANA data: overall mean difference in systolic blood pressure under different meta‐analysis models

M‐A model	Estimation procedure	$\hat{\beta }$ (95% CI), mm Hg
Fixed effect, shared $\sigma_{i}^{2}$	One‐stage	−12.5254 (−12.9128, −12.1382)
	Two‐stage	−12.5255 (−12.9112, −12.1398)
Fixed effect, study‐specific $\sigma_{i}^{2}$	One‐stage^a^	−12.3404 (−12.7236, −11.9571)
	Two‐stage^a^	−12.3404 (−12.7236, −11.9571)
Random effects, study‐specific $\sigma_{i}^{2}$	One‐stage	−15.2069 (−19.3984, −11.0153)
(REML with Kenward–Roger adjustment)	Two‐stage	−15.2068 (−18.5534, −11.8602)

^a^Confidence intervals (CIs) computed using asymptotic variance formula (1).

## 5 DISCUSSION

Focusing on practical sample sizes (rather than asymptotic results), this paper attempts to resolve the debate about using one‐ or two‐stage estimation methods in meta‐analysis. We have considered Gaussian data and demonstrated theoretically (for fixed effect) and via simulation (for random‐effects models), that provided the same underlying model is used, inference from one‐ and two‐stage procedures is practically equivalent for 2 models which are practically relevant.

To make progress, we focused on a very simple data structure and considered combining evidence from randomised studies. We considered the main arguments regarding “flexibility” and precision. Table 1 shows that a wider range of statistical models can be fitted in a one‐stage framework; however, for many meta‐analyses, this flexibility is not needed.

To study precision, we derived some new results. Specifically, the one‐stage fixed‐effects estimator of *β* which allows for study‐specific variances; a new variance estimator which admits that ${\hat{\sigma }}_{i}^{2}$ are estimated and not known (again for the fixed‐effect model allowing study‐specific variances); and estimated the expectation of this formula, comparing it to the expectation of the OLS variance, where ${\hat{\sigma }}_{i}^{2}$ is assumed to be equal across all studies. For random‐effect meta‐analysis, we also calculated and implemented the Kenward–Roger small sample correction.

In practical sample sizes, our work shows that the arguments regarding precision are largely redundant, as previously shown asymptotically for more general models.^7^ Mathew and Nördstrom claim to show that a one‐stage analysis will always be at least as precise as two‐stage, but the only example of an inequality is for a meta‐analysis with no study effects at all, which is of no practical interest.^6^ Appendix A.1 shows that the fixed‐effect model can be fitted using weighted one‐stage estimation, which returns the same estimates as would be obtained through inverse‐variance weighted two‐stage estimation. Meanwhile, Senn^15^ shows how OLS estimates can be recovered from summary data in two stages. The results displayed in Figure 1 show that the latter is more precise than the former. Thus, analysis in two stages may be less precise, more precise, or identical to two stages, depending on the modelling assumptions made by the computational procedure. In summary, these papers do not contradict our key finding, which is that for equivalent models, fitting by a one‐ or two‐stage procedure gives practically equivalent results.

Improvements in precision must be attributed to different modelling assumptions, that is, the precision argument for one‐stage procedures is actually about *flexibility* again. A different model may be more or less precise: In general, we buy information with assumptions. We argue that model choice should never be based on precision. Opting to fit a model in one stage or two based on the perceived efficiency is in fact doing exactly this. In practice, it is important to make modelling assumptions that are tenable and fit the model as using whatever procedure allows for these assumptions. For the assumptions which may change between one‐ and two‐stage procedures, see Burke et al.^9^

Due to the simple data structure considered, we have not touched on covariate adjustment here. Adjusting for the main effects of covariates may be desirable, either to obtain a valid estimate of $\mathrm{Var}({\hat{\beta }}_{i})$(for example, where covariates have been balanced by design^25^ or to increase power.^26^ Here, a one‐stage model can estimate covariate effects to have the same values across all studies. Two‐stage analysis relaxes this assumption by fitting a model which implies separate covariate effects for each study, which we conjecture is the correct way to ensure a valid estimate of $\mathrm{Var}({\hat{\beta }}_{i})$. To fit a one‐stage version of this model, covariate–study interaction terms need to be included and, if the modelling assumptions are identical, our results would hold. If the covariate effects truly differ by study, then the model that incorporates this (whether fitted by a one‐ or two‐stage procedure) will tend to give more precise estimates; the converse is also true.

For meta‐analysis of patient‐level treatment‐covariate interactions, using the two‐stage procedure of fitting a model including interactions and combining these within‐study interactions in a second‐stage meta‐analysis, guards against ecological bias.^27^, ^28^, ^29^ As always, a practically equivalent model can be fitted using a one‐stage procedure; however, in this setting, care needs to be taken over the parameterisation and study‐specific covariate means must be adjusted for.^28^, ^29^ This may not be intuitive, and in this context, a one‐stage analysis should aim to estimate the same effect as a two‐stage analysis. Here, a model that can be fitted in one stage but not two may actually be misleading (“deluded” in the parlance of Fisher et al).^28^

For practical meta‐analysis, a clear description of the model and assumptions is important. See Burke et al^9^ for a description of practical assumptions which may differ between one‐ and two‐stage meta‐analysis: The relevance of each of these should be considered and described.

When fitting a model in two stages, it is important to describe whether a fixed‐ or random‐effects model is being used. For fixed‐effect models, the assumption about common or study‐specific ${\hat{\sigma }}_{i}^{2}$ then needs to be described. For random effects, the random‐effects model should be described. The clearest way of doing this is in terms of the estimator of tau squared (eg, DerSimonian–Laird or REML^3^ followed by any additional details such as use of the Hartung–Knapp/Sidik–Jonkman or Kenward–Roger variance correction.

When fitting a model in one stage, some modelling aspects to describe may be whether random effects were estimated and the parameters they were attached to; whether ${\hat{\sigma }}_{i}^{2}$ was shared or study specific; any correction to the covariance matrix; whether parameters for covariate effects were constrained to be equal or allowed to vary across studies; and how interactions were estimated.

We encourage those involved in meta‐analysis methodology to focus more on consideration of models and assumptions and less on the procedures used for parameter estimation. The procedure used (one or two stages) is a computational tool; the model being fitted is what the tool aims to construct, and a tool that does the required job conveniently should be favoured.

## 6 Data Accessibility

## APPENDIX A PROPERTIES OF WEIGHTED ESTIMATORS

The below sections derive the variance of the fixed‐effects inverse‐variance estimator. Unless otherwise stated, summations are over all *i*, the study index.

### A1 Variance estimator for a one‐stage analysis assuming varying variances

The fixed effects model is *Y*_*i**j*_=*α*_*i*_+*β* *X*_*i**j*_+*ε*_*i**j*_, where *ε*_*i**j*_∼N(0,*σ* ^2^), *i* indexes studies and *j* indexes patients. This model is estimated by (*X* ^*′*^ *X*)^−1^ *X* ^*′*^ *Y* and covariance matrix by ${\left(X^{\prime }X\right)}^{-1}{\hat{\sigma }}^{2}$. We require the expectation of $\hat{\mathrm{Var}}(\hat{\beta })$.

Assume that we have *I* studies, each with *n*_*i*_ patients. Define the $\sum n_{i}\times (I+1)$ design matrix

$$X=\left(\begin{array}{ccccc} 1 & 0 & \text{⋯} & 0 & 1\\ \vdots & & & & \vdots \\ 1 & 0 & \text{⋯} & 0 & 0\\ 0 & 1 & \text{⋯} & 0 & 1\\ \vdots & & & & \vdots \\ 0 & 1 & \text{⋯} & 0 & 0\\ \vdots & & & & \vdots \\ 0 & 0 & \text{⋯} & 1 & 1\\ \vdots & & & & \vdots \\ 0 & 0 & \text{⋯} & 1 & 0 \end{array}\right).$$

The first *I* columns indicate (exclusive) membership in study *i*=1,…,*I* and the (*I*+1)th indicates treatment assignments. For a one‐stage analysis, $\hat{\mathrm{Var}}(\hat{\beta })$ is the (*I*+1)th diagonal element of ${\hat{\sigma }}^{2}{\left(X^{\prime }X\right)}^{-1}$. First, (***X*** *′* ***X***) is (*I*+1)×(*I*+1):

$$X^{\prime }X=\left(\begin{array}{cccc} n_{1} & 0 & \text{⋯} & n_{1}/2\\ 0 & n_{2} & & n_{2}/2\\ \vdots & & \ddots & \vdots \\ n_{1}/2 & n_{2}/2 & \text{⋯} & \sum n_{i}/2 \end{array}\right).$$

The inverse is

$${\left(X^{\prime }X\right)}^{-1}=\left(\begin{array}{cccc} \; & \; & \text{⋯} & -2/\sum n_{i}\\ \; & \; & & -2/\sum n_{i}\\ \vdots & & \ddots & \vdots \\ -2/\sum n_{i} & -2/\sum n_{i} & \text{⋯} & 4/\sum n_{i} \end{array}\right).$$

We are interested in the last element (variance of the treatment effect), which is $\hat{\mathrm{Var}}(\hat{\beta })=\frac{4\sigma^{2}}{\sum n_{i}}$.

The shared‐*σ* ^2^ assumption can be relaxed in the following way: Construct a $\sum n_{i}\times \sum n_{i}$ diagonal matrix of weights *W*, where the nonzero elements are $1/{\hat{\sigma }}_{i}^{2}$; the variance–covariance matrix is then estimated as ${\left(X^{\prime }WX\right)}^{-1}{\hat{\sigma }}^{2}$. The matrix *X* *′* *W* *X* is

The solid lines show how the matrix can be partitioned to invert the (*I*+1)th diagonal element (variance of the treatment effect), which is

$${\left(\sum \frac{n_{i}}{4\sigma_{i}^{2}}\right)}^{-1}.$$

This is identical to the standard inverse‐variance formula used by a two‐stage procedure. The algebra can also be worked through for the estimator of *β* to include weights *W*, that is (*X* *′* *W* *X*)^−1^ *X* *′* *W* *y*. The one‐ and two‐stage procedures then give identical results for $\hat{\beta }$. This complements the result described and worked through in Senn,^15^ taken from Olkin and Sampson,^5^ which shows that a two‐stage procedure can obtain one‐stage estimates when modified to make the same assumptions of equal $\sigma_{i}^{2}\forall i$.

### A2 Expected value of standard two‐stage variance estimator

Let *i*=1,…,*I* index studies and *j*=1,…,*n*_*i*_ index individuals. The unadjusted estimator of *β* for active vs control is

$${\hat{\beta }}_{i}=\frac{2}{n_{i}}\sum_{\text{actv.}}y_{j}-\frac{2}{n_{i}}\sum_{\text{cont.}}y_{j}$$

with variance for study *i* estimated by

$$\hat{\mathrm{Var}}({\hat{\beta }}_{i})=\frac{4{\hat{\sigma }}_{i}^{2}}{n_{i}}.$$

This first stage is repeated within each study. Define weights ${\hat{w}}_{i}=n/4{\hat{\sigma }}_{i}^{2}$ and let

$$\hat{W}=\sum_{i=1}^{I}{\hat{w}}_{i}.$$

For the second stage, the inverse‐variance estimator of the overall treatment effect is

(A1) $$\hat{\beta }=\frac{\sum {\hat{w}}_{i}{\hat{\beta }}_{i}}{\hat{W}}.$$

The variance of *β* is almost always estimated by

(A2) $$\hat{\mathrm{Var}}(\hat{\beta })=\frac{1}{\hat{W}}=\sum_{i=1}^{I}\frac{4{\hat{\sigma }}_{i}^{2}}{n_{i}}.$$

We now calculate the expectation of $\hat{\mathrm{Var}}(\hat{\beta })$ for 2 studies. Note that

(A3) $$\begin{array}{ccc} {\hat{\sigma }}_{i}^{2} & ∼\frac{\chi_{n_{i}-2}^{2}\sigma_{i}^{2}}{n_{i}-2}, & \\ \text{so}\;\;E[{\hat{\sigma }}_{i}^{2}] & =\sigma_{i}^{2},\\ \text{and}\;\;\mathrm{Var}({\hat{\sigma }}_{i}^{2}) & =\frac{2\sigma_{i}^{4}}{n_{i}-1}.\\ \text{Write}\;\;{\hat{\sigma }}_{i}^{2} & ∼\sigma_{i}^{2}+\varepsilon_{i},\\ \text{where}\;\;\varepsilon_{i} & ∼\left(0,\frac{2\sigma_{i}^{4}}{n_{i}-1}\right). \end{array}$$

For a meta‐analysis of two studies, our variance estimator is

$$\hat{\mathrm{Var}}(\hat{\beta })=4{\left(n_{1}{\hat{\sigma }}_{1}^{-2}+n_{2}{\hat{\sigma }}_{2}^{-2}\right)}^{-1}.$$

Substituting $x={\hat{\sigma }}_{1}^{2},y={\hat{\sigma }}_{2}^{2}$, write $\hat{\mathrm{Var}}(\hat{\beta })$ as

$$f(x,y)=4{\left(n_{1}x^{-1}+n_{2}y^{-1}\right)}^{-1}.$$

The first and second partial derivatives for *x* and *y* are

$$\begin{array}{ccc} \frac{\partial f}{\partial x} & =n_{1}x^{-2}f^{2}, & \\ \frac{\partial f}{\partial y} & =n_{2}y^{-2}f^{2},\\ \frac{\partial_{2}f}{\partial x^{2}} & =-2n_{1}x^{-3}f^{2}+2n_{1}^{2}x^{-4}f^{3},\\ \frac{\partial_{2}f}{\partial y^{2}} & =-2n_{2}y^{-3}f^{2}+2n_{2}^{2}y^{-4}f^{3}. \end{array}$$

Denote *f*_*x*_ after evaluating $\frac{\partial f}{\partial x}$ at $\mu_{x}=\sigma_{1}^{2},\mu_{y}=\sigma_{y}^{2}$, and similarly for *f*_*y*_,*f*_*x**x*_,*f*_*y**y*_. By a Taylor expansion,

$$\begin{array}{ccc} f(x,y)≃ & f(\mu_{x},\mu_{y})+(\varepsilon_{x},\varepsilon_{y})\left(\begin{array}{c} f_{x}\\ f_{y} \end{array}\right)+\frac{1}{2}(\varepsilon_{x},\varepsilon_{y})\left(\begin{array}{cc} f_{xx} & f_{xy}\\ f_{xy} & f_{yy} \end{array}\right)\left(\begin{array}{c} \varepsilon_{x}\\ \varepsilon_{y} \end{array}\right) & \\ = & f(\mu_{x},\mu_{y})+\varepsilon_{x}f_{x}+\varepsilon_{y}f_{y}+\frac{1}{2}\left(\varepsilon_{x}^{2}f_{xx}+2\varepsilon_{x}\varepsilon_{y}f_{xy}+\varepsilon_{y}^{2}f_{yy}\right). \end{array}$$

Note that E[*ε*_*x*_]=E[*ε*_*y*_]=0, and studies are uncorrelated, so E[*ε*_*x*_ *ε*_*y*_]=0, then

(A4) $$E[\mathrm{Var}(\hat{\beta })]≃f(\mu_{x},\mu_{y})+\frac{1}{2}(E[\varepsilon_{x}^{2}]f_{xx})+\frac{1}{2}(E[\varepsilon_{y}^{2}]f_{yy}).$$

Within each study,

$$E[\varepsilon_{i}^{2}]=\frac{2\sigma_{i}^{4}}{n_{i}-1}.$$

Generalising to *I* studies,

$$\begin{array}{ccc} f(x_{1},\text{⋯},x_{I}) & =4{\left(n_{1}x_{1}^{-1}+\text{⋯}+n_{I}x_{I}^{-1}\right)}^{-1} & \\ f_{x_{i}x_{i}} & =2(n_{i}x_{i}^{-4}f^{3}-n_{i}x_{i}^{-3}f^{2}). \end{array}$$

This gives us that

(A5) $$\begin{array}{ccc} E[\hat{\mathrm{Var}}(\hat{\beta })] & ≃f(\sigma_{1}^{2},\text{⋯},\sigma_{I}^{2})+\frac{1}{2}\sum_{i=1}^{I}E[\varepsilon_{i}^{2}]f_{\sigma_{i}^{2},\sigma_{i}^{2}} & \\ & =4{\left(\sum_{i=1}^{I}n_{i}\sigma_{i}^{-2}\right)}^{-1}+\frac{1}{2}\sum_{i=1}^{I}\left(\frac{2\sigma_{i}^{2}}{n_{i}-1}f_{\sigma_{i}^{2}\sigma_{i}^{2}}\right). \end{array}$$

This result is used to compare the expectation of (A2) to the OLS fixed‐effect estimator (results given in Figure 1, upper panel).

### A3 An improved variance estimator to partner the inverse‐variance estimator of $\hat{\beta }$

Because the standard variance formula given in (A2) is biased downwards in finite samples, we derive an improved estimator for the variance of (A1). Let

$$\hat{\beta }=f({\hat{\beta }}_{1},{\hat{\sigma }}_{1},\text{⋯},{\hat{\beta }}_{I},{\hat{\sigma }}_{I})=\frac{\sum {\hat{w}}_{i}{\hat{\beta }}_{i}}{\hat{W}}.$$

First derivatives wrt ${\hat{\beta }}_{i}$ and ${\hat{w}}_{i}$ are

$$\begin{array}{ccc} \frac{\partial f}{\partial {\hat{w}}_{i}} & =\frac{\beta_{i}}{\hat{W}}-\frac{\hat{\beta }}{\hat{W}}, & \\ \frac{\partial f}{\partial {\hat{\beta }}_{i}} & =\frac{{\hat{w}}_{i}}{\hat{W}}. \end{array}$$

By a Taylor expansion of *f*(), we get

(A6) $$\begin{array}{cc} \mathrm{Var}(\hat{\beta })≃ & \left(\frac{\partial f}{\partial {\hat{\beta }}_{1}},\frac{\partial f}{\partial {\hat{w}}_{1}},\text{⋯},\frac{\partial f}{\partial {\hat{\beta }}_{I}},\frac{\partial f}{\partial {\hat{w}}_{I}}\right)\times \left(\begin{array}{ccccc} \frac{4{\hat{\sigma }}_{1}^{2}}{n_{1}} & 0 & \text{⋯} & 0 & 0\\ 0 & \frac{2{\hat{\sigma }}_{1}^{4}}{n_{1}-1} & & 0 & 0\\ \vdots & & \ddots & & \vdots \\ 0 & 0 & & \frac{4{\hat{\sigma }}_{I}^{2}}{n_{I}} & 0\\ 0 & 0 & \text{⋯} & 0 & \frac{2{\hat{\sigma }}_{I}^{4}}{n_{I}-1} \end{array}\right)\times \left(\begin{array}{c} \frac{\partial f}{\partial {\hat{\beta }}_{1}}\\ \frac{\partial f}{\partial {\hat{w}}_{1}}\\ \vdots \\ \frac{\partial f}{\partial {\hat{\beta }}_{I}}\\ \frac{\partial f}{\partial {\hat{w}}_{I}} \end{array}\right) \end{array}$$

(A7) $$\begin{array}{ccc} = & \sum \frac{1}{{\hat{w}}_{i}}{\left(\frac{{\hat{w}}_{i}}{\hat{w}}\right)}^{2}+\sum \frac{2\overset{4}{\underset{i}{\sigma }}}{n_{i}-1}{\left(\frac{{\hat{\beta }}_{i}-\hat{\beta }}{\hat{W}}\right)}^{2} & \\ = & \frac{1}{\hat{W}}+\sum_{i=1}^{2}\frac{2\sigma_{i}^{4}}{n_{i}-1}{\left(\frac{{\hat{\beta }}_{i}-\hat{\beta }}{\hat{W}}\right)}^{2}. \end{array}$$

The bold **0**s in (A6) are always equal to 0 in meta‐analysis provided studies can be assumed independent. The roman 0s are set to 0 here because we are considering linear models, where an estimate and its variance are independent. Note that when *β*_*i*_ and Var(*β*_*i*_) cannot be assumed independent, as with binary outcomes, the roman 0s in (A6) need to be replaced with the correct terms.

(A7) provides a variance estimator for Gaussian data which improves on (A2) in finite samples.

### A4 Expected value of the variance estimator (A7)

The estimator is given by A1, where

$$\begin{array}{ccc} {\hat{w}}_{i} & =\frac{n_{i}}{4{\hat{\sigma }}_{i}^{2}}∼\frac{n_{i}v_{i}X_{i}}{4\sigma_{i}^{2}} & \\ \text{for}\;\;X_{i}^{-1} & ∼\chi_{v_{i}}^{2},\\ \text{and}\;\;{\hat{\beta }}_{i} & ∼N\left(\beta ;\frac{4\sigma_{i}^{2}}{n_{i}}\right). \end{array}$$

${\hat{\beta }}_{i}$ and ${\hat{w}}_{i}$ are assumed to be independent. Note that ${\hat{w}}_{i}$ can be expressed as a function of the random variable *X*_*i*_. Let *X* represent the set of all *X*_*i*_s.

We want the variance of $\hat{\beta }$.

$$\begin{array}{ccc} \mathrm{Var}(\hat{\beta }) & =E_{X}\left\{\mathrm{Var}(\hat{\beta }|X)\right\}+{\mathrm{Var}}_{X}\left\{\left.E({\hat{\beta }}_{i})|X\right)\right\} & \\ & =E_{X}\left\{\frac{{\hat{w}}_{i}^{2}\frac{4\sigma_{i}^{2}}{n_{i}}}{{\left(\sum {\hat{w}}_{i}\right)}^{2}}\right\}+{\mathrm{Var}}_{X}(\beta )\\ & =E_{X}\left\{\frac{A}{B}\right\},\\ \text{for}\;\;A & =\sum e_{i}X_{i}^{2}\\ \text{and}\;\;B & ={\left(\sum f_{i}X_{i}\right)}^{2},\\ \text{where}\;\;e_{i} & =\frac{n_{i}v_{i}^{2}}{4\sigma_{i}^{2}}\\ \text{and}\;\;f_{i} & =\frac{n_{i}v_{i}}{4\sigma_{i}^{2}}=\frac{e_{i}}{v_{i}}. \end{array}$$

The generic problem is that we want the expectation of the ratio

$$R=\frac{A}{B}$$

for *A* and *B* as defined above with arbitrary positive constants *e*_*i*_ and *f*_*i*_.

Use the second‐order expression^30^

(A8) $$E(R)≃\frac{E(A)}{E(B)}-\frac{\text{Cov}(A,B)}{E{\left(B\right)}^{2}}+\frac{\mathrm{Var}(B)E(A)}{E{\left(B\right)}^{3}}.$$

The calculation of the required moments follows.

$$\begin{array}{ccc} E(A) & =\sum_{i}\frac{e_{i}}{\left(v_{i}-2)(v_{i}-4\right)}, & \\ \; & \\ E(B) & =E_{X}\left\{{\left(\sum_{i}f_{i}X_{i}\right)}^{2}\right\}\\ & =E\left(\sum_{i}\sum_{j}f_{i}f_{j}X_{i}X_{j}\right)\\ & =\sum_{i}f_{i}^{2}E(X_{i}^{2})+\sum_{i\neq }\sum_{j}f_{i}f_{j}E(X_{i})E(X_{j})\\ & =\sum_{i}\frac{f_{i}^{2}}{\left(v_{i}-2)(v_{i}-4\right)}+\sum_{i\neq }\sum_{j}f_{i}f_{j}\frac{1}{\left(v_{i}-2)(v_{j}-2\right)},\\ \text{Cov}(A,B) & =\text{Cov}\left\{\sum e_{i}X_{i}^{2},{\left(\sum f_{i}X_{i}\right)}^{2}\right\}\\ & =\sum_{i}\sum_{j}\sum_{k}e_{i}f_{j}f_{k}\text{Cov}(X_{i}^{2},X_{j}X_{k})\\ & =\sum_{i}e_{i}f_{i}^{2}\mathrm{Var}(X_{i}^{2})+\sum_{i\neq }\sum_{j}2e_{i}f_{i}f_{j}\text{Cov}(X_{i}^{2},X_{i}X_{j}), \end{array}$$

$$\begin{array}{ccc} \text{and}\;\;\mathrm{Var}(X_{i}^{2}) & =\frac{8(v_{i}-5)}{{\left(v_{i}-2\right)}^{2}{\left(v_{i}-4\right)}^{2}(v_{i}-6)(v_{j}-2)}, & \\ \; & \\ \text{Cov}(X_{i}^{2},X_{i}X_{j}) & =\frac{4}{{\left(v_{i}-2\right)}^{2}(v_{i}-4)(v_{i}-6)(v_{j}-2)}, \end{array}$$

$$\begin{array}{ccc} E(B^{2}) & =E\left\{{\left(\sum_{i}f_{i}X_{i}\right)}^{4}\right\} & \\ & =E\left(\sum_{i}\sum_{j}\sum_{k}\sum_{l}f_{i}f_{j}f_{k}f_{l}X_{i}X_{j}X_{k}X_{l}\right)\\ & =\sum_{i}f_{i}^{4}E(X_{i}^{4})+4\sum_{i\neq }\sum_{j}f_{i}^{3}f_{j}E(X_{i}^{3}X_{j})\\ & \;+3\sum_{i\neq }\sum_{j}f_{i}^{2}f_{j}^{2}E(X_{i}^{2})E(X_{j}^{2})\\ & \;+6\sum_{i\neq }\sum_{j\neq }\sum_{k}f_{i}^{2}f_{j}f_{k}E(X_{i}^{2})E(X_{j})E(X_{k})\\ & \;+\sum_{i\neq }\sum_{j\neq }\sum_{k\neq }\sum_{l}f_{i}f_{j}f_{k}f_{l}E(X_{i})E(X_{j})E(X_{k})E(X_{l})\\ & =\sum_{i}f_{i}^{4}\frac{1}{\left(v_{i}-2)(v_{i}-4)(v_{i}-6)(v_{i}-8\right)}\\ & \;+4\sum_{i\neq }\sum_{j}f_{i}^{3}f_{j}\frac{1}{\left(v_{i}-2)(v_{i}-4)(v_{i}-6)(v_{j}-2\right)}\\ & \;+3\sum_{i\neq }\sum_{j}f_{i}^{2}f_{j}^{2}\frac{1}{\left(v_{i}-2)(v_{i}-4)(v_{j}-2)(v_{j}-4\right)}\\ & \;+6\sum_{i\neq }\sum_{j\neq }\sum_{k}f_{i}^{2}f_{j}f_{k}\frac{1}{\left(v_{i}-2)(v_{i}-4)(v_{j}-2)(v_{k}-2\right)}\\ & \;+\sum_{i\neq }\sum_{j\neq }\sum_{k\neq }\sum_{l}f_{i}f_{j}f_{k}f_{l}\frac{1}{\left(v_{i}-2)(v_{j}-2)(v_{k}-2)(v_{l}-2\right)}.\\ \text{Then,}\;\;\mathrm{Var}(B) & =E(B^{2})-E{\left(B\right)}^{2}. \end{array}$$

Substituting the expectations E(*A*), E(*B*), Cov(*A*,*B*), and Var(*B*) into (A8) gives an expression for the actual variance of $\hat{\beta }$.

This has been verified in simulation; the Mata code to calculate the formula and verify (A8) and its components is provided as a supplement. Also included is the log file. This is run for 7 studies where, arbitratily, *f*=[1,2,3,4,5,6,7] and $e=[10,5,3\frac{1}{3},2.5,2,1\frac{2}{3},1\frac{3}{7}]$. We simulated the *X*_*i*_s from $1/\chi_{v_{i}}^{2}$ and calculated

$$\begin{array}{ccc} A & =\sum_{i=1}^{7}e_{i}X_{i}^{2}, & \\ B & ={\left(\sum_{i=1}^{7}f_{i}X_{i}\right)}^{2},\\ \text{and}\;\;R & =\;\frac{A}{B}. \end{array}$$

We provide 2 simulation results. First, Var(*R*) is estimated empirically from the simulated values; second, it is calculated using (A8) where empirical values of E(*A*), E(*B*), Var(*B*), and Cov(*A*,*B*) are substituted for the theoretical expectations. These results, and those based on the expectations, are given in Table A1 for 1×10^10^ repetitions. A tiny error in the approximation (A8) is seen, but the empirical and theoretical components of (A8) are near identical. This justifies the use of the theoretical formula to compare expected variances of the inverse‐variance and OLS estimators (as in Figure 1).

**Table A1:** Simulation‐based check of variance formula (1×10^10^ repetitions)

	Empirical	Approximate empirical,	Theoretical
		based on (A8)	based on (A8)
E(*A*)	…	0.02695	0.02695
E(*B*)	…	1.17552	1.17554
Var(*B*)	…	0.11525	0.11523
Cov(*A* *B*)	…	0.00157	0.00157
Var(*A*/*B*)	**0.023618**	**0.023706**	**0.023704**
Ratio vs empirical	1	1.00374	1.00364

## APPENDIX B KENWARD–ROGER CORRECTION FOR TWO‐STAGE RANDOM‐EFFECTS META‐ANALYSIS ESTIMATED BY REML

This section derives the Kenward–Roger variance estimator and degrees of freedom for two‐stage meta‐analysis using the notation of Kenward and Roger.^19^, ^31^) We first recap the general approach and notation before applying it to the meta‐analysis model.

The general linear mixed‐effects model can be written as

y=Xβ+Zγ+e,

where ***y*** is a (*k*×1) vector of random variables, ***X*** is a (*k*×*p*) matrix of known constants for the (*p*×1) fixed‐effect parameter vector ***β***, ***Z*** is the (*k*×*q*) design matrix for the (*q*×1) random‐effects parameter vector ***γ***, and ***e*** is a (*k*×1) vector of random error terms.

We assume E[***γ***]=0, E[***e***]=0, and Cov[***γ***,***e***]=0. Define ***D*** as the (*q*×*q*) covariance matrix of the random‐effects parameters in ***γ***, and ***R*** as the (*k*×*k*) covariance matrix of ***e***. Then, **Σ**, the (*k*×*k*) covariance matrix of ***y***, is equal to ***Z**D**Z*** ^⊤^+***R***. After assuming normality of the random terms in the model, we obtain***y***∼N(***X*** ***β***,**Σ**).

Kenward and Roger describe **Σ** under the general linear mixed‐effects model as being an unknown, block diagonal, (*k*×*k*) variance‐covariance matrix whose elements are well‐behaved functions of ***σ***(*r*×1).^19^ The standard approach would be to use REML to find an estimate $\hat{\sigma }$ for ***σ*** and proceed to find a REML estimator $\hat{\beta }=\Phi (\hat{\sigma })X^{⊤}\Sigma {\left(\hat{\sigma }\right)}^{-1}Y$ for ***β***, with variance $\hat{\Phi }(\hat{\sigma })={\left[X^{⊤}\Sigma {\left(\hat{\sigma }\right)}^{-1}X\right]}^{-1}$, where **Σ** and hence **Φ** are dependent on $\hat{\sigma }$.

The standard REML estimator of $\mathrm{Var}(\hat{\beta })$, $\hat{\Phi }={\left[X^{⊤}\Sigma^{-1}X\right]}^{-1}$, is the variance‐covariance matrix of the asymptotic limiting distribution of $\hat{\beta }$ based on the REML estimation of ***σ***. Kenward and Roger obtain a better approximation than $\hat{\Phi }$ to the finite‐sample variance‐covariance matrix of $\hat{\beta }$.

The corrected estimator can be written as

$${\hat{\Phi }}_{A}=\hat{\Phi }+2\hat{\Phi }\left\{\sum_{i=1}^{r}\sum_{j=1}^{r}W_{ij}\left(Q_{ij}-P_{i}\hat{\Phi }P_{j}-\frac{1}{4}R_{ij}\right)\right\}\hat{\Phi }-\frac{1}{4}\sum_{s,t=1}^{r}W_{st}V_{t}\hat{\Phi }P_{s}\hat{\Phi },$$

where

$$\begin{array}{ccc} P_{i} & =X^{⊤}\frac{\partial \Sigma^{-1}}{\partial \sigma_{i}}X, & \\ Q_{ij} & =X^{⊤}\frac{\partial \Sigma^{-1}}{\partial \sigma_{i}}\Sigma \frac{\partial \Sigma^{-1}}{\partial \sigma_{j}}X,\\ R_{ij} & =X^{⊤}\Sigma^{-1}\frac{\partial^{2}\Sigma }{\partial \sigma_{i}\partial \sigma_{j}}\Sigma^{-1}X, \end{array}$$

and *W*_*i**j*_ is the (*i*,*j*)th element of the variance‐covariance matrix of ***σ***, that is, of ***W***=Var[***σ***], which may be approximated by the inverse of the observed or expected information matrix. Kenward and Roger give the following expression for the expected information:

$$I{\left(\hat{\sigma }\right)}_{ij}=\frac{1}{2}\text{tr}\left(\frac{\partial \Sigma^{-1}}{\partial \sigma_{i}}\Sigma \frac{\partial \Sigma^{-1}}{\partial \sigma_{j}}\Sigma \right)-\frac{1}{2}\text{tr}\left(2\hat{\Phi }Q_{ij}-\hat{\Phi }P_{i}\hat{\Phi }P_{j}\right).$$

The third term of ${\hat{\Phi }}_{A}$, and therefore *V*_*i*_ and ***S***_*i**j*_, may be ignored if the covariance matrix **Σ** is linear in ***σ***. Those quantities are defined as follows:

$$\begin{array}{ccc} V_{i} & =\text{tr}\left\{S\frac{\partial \Sigma }{\partial \sigma_{i}}\right\}-2\text{tr}\left\{\left(X^{⊤}\Sigma^{-1}\frac{\partial \Sigma }{\partial \sigma_{i}}SX\right)\hat{\Phi }\right\}+\text{tr}\left\{\left(X^{⊤}SX\right)\hat{\Phi }\left(X^{⊤}\Sigma^{-1}\frac{\partial \Sigma }{\partial \sigma_{i}}\Sigma^{-1}X\right)\hat{\Phi }\right\}, & \\ S_{ij} & =\Sigma^{-1}\left(\sum_{i,j=1}^{r}W_{ij}\frac{\partial^{2}\Sigma }{\partial \sigma_{i}\partial \sigma_{j}}\right)\Sigma^{-1}. \end{array}$$

### B1 The random‐effects meta‐analysis model

For the random‐effects meta‐analysis model, ***y*** consists of the *I* treatment effect estimates *β*_*i*_, ***X***=**1**_*i*_, ***β*** includes the “pooled” effect size *β*, and **Σ** is diagonal with elements $\Sigma_{ii}={\hat{\sigma }}_{i}^{2}+{\hat{\tau }}^{2}$, where ${\hat{\sigma }}_{i}^{2}$ is the estimated variance of *β*_*i*_ and ${\hat{\tau }}^{2}$ is the estimated between‐study heterogeneity.

We proceed by noting that Kenward and Roger's *σ*_1_=*τ* ^2^(note that this is distinct from *σ*_*i*_, the within‐study standard deviation) and also define $w_{i}={\left(\sigma_{i}^{2}+\sigma_{1}\right)}^{-1}$, so that $\Sigma_{ii}=w_{i}^{-1}$. Then,

$$\begin{array}{ccc} \hat{\mathrm{Var}}(\hat{\beta })=\hat{\Phi } & =\frac{1}{\sum_{i}w_{i}}, & \\ {\left(\frac{\partial \Sigma^{-1}}{\partial \sigma_{1}}\right)}_{ii} & =-w_{i}^{2},\\ P_{1} & =-\sum_{i}w_{i}^{2},\\ Q_{11} & =\sum_{i}w_{i}^{3}. \end{array}$$

Note that **Σ** is linear in *σ*_1_, so that $\frac{\partial^{2}\Sigma }{\partial \sigma_{1}^{2}}=0$. This immediately implies that ***R***_11_=0 and also that the entire third term of the above expression for ${\hat{\Phi }}_{A}$ is 0.

Then,

$$\begin{array}{ccc} Q_{11}-P_{1}\hat{\Phi }P_{1} & =\sum_{i}w_{i}^{3}-\frac{{\left(\sum_{i}w_{i}^{2}\right)}^{2}}{\sum_{i}w_{i}} & \\ \Rightarrow {\hat{\Phi }}_{A} & =\frac{1}{\sum_{i}w_{i}}+\frac{2W_{11}}{{\left(\sum_{i}w_{i}\right)}^{2}}\left[\sum_{i}w_{i}^{3}-\frac{{\left(\sum_{i}w_{i}^{2}\right)}^{2}}{\sum_{i}w_{i}}\right]\\ & =\frac{1}{\sum_{i}w_{i}}\left\{1+2W_{11}\left(\frac{\sum_{i}w_{i}^{3}}{\sum_{i}w_{i}}-{\left[\frac{\sum_{i}w_{i}^{2}}{\sum_{i}w_{i}}\right]}^{2}\right)\right\}, \end{array}$$

where *W*_11_=Var[*σ*_1_] as explained earlier.

The expected information works out to be

$$I_{E}({\hat{\sigma }}_{1})=\frac{1}{2}\sum_{i}w_{i}^{2}-\frac{\sum_{i}w_{i}^{3}}{\sum_{i}w_{i}}+\frac{1}{2}{\left[\frac{\sum_{i}w_{i}^{2}}{\sum_{i}w_{i}}\right]}^{2}.$$

If we wish to use the observed information, this requires the second derivative of the log‐likelihood with respect to *σ*_1_.

$$\log L(\sigma_{1})=\text{constant}+\frac{1}{2}\sum_{i}\log(w_{i})-\frac{1}{2}\log\sum_{i}w_{i}-\frac{1}{2}\sum_{i}w_{i}{\left(\beta_{i}-\hat{\beta }\right)}^{2}$$

Note that $\hat{\beta }$ is itself a function of *σ*_1_ and must also be differentiated. Concentrating first on this term, let $v_{i}=\beta_{i}-\hat{\beta }$ so that the final term becomes $-\frac{1}{2}\sum_{i}w_{i}v_{i}^{2}$. Then,

$$-\frac{1}{2}\frac{\partial }{\partial \sigma_{1}}\sum_{i}w_{i}v_{i}^{2}=-\sum_{i}w_{i}v_{i}v_{i}^{\prime }-\frac{1}{2}\sum_{i}w_{i}^{\prime }v_{i}^{2}.$$

Now, using the quotient rule and rearranging,

$$\begin{array}{ccc} v_{i}^{\prime }=\frac{\partial }{\partial \sigma_{1}}\frac{\sum w_{i}\beta_{i}}{\sum w_{i}} & =-\frac{\sum_{i}w_{i}^{2}v_{i}}{\sum_{i}w_{i}}\;\forall i & \\ \Rightarrow -\sum_{i}w_{i}v_{i}v_{i}^{\prime } & =\frac{\sum_{i}w_{i}^{2}v_{i}}{\sum_{i}w_{i}}\sum_{i}w_{i}v_{i}.\\ \text{But}\;\sum_{i}w_{i}v_{i} & =\sum_{i}w_{i}\beta_{i}-\sum_{i}w_{i}\frac{\sum_{i}w_{i}\beta_{i}}{\sum_{i}w_{i}}=0\\ \Rightarrow -\frac{1}{2}\frac{\partial }{\partial \sigma_{1}}\sum_{i}w_{i}v_{i}^{2} & =-\frac{1}{2}\sum_{i}w_{i}^{\prime }v_{i}^{2}=\frac{1}{2}\sum_{i}w_{i}^{2}v_{i}^{2}\\ \Rightarrow -\frac{1}{2}\frac{\partial^{2}}{\partial \sigma_{1}^{2}}\sum_{i}w_{i}v_{i}^{2} & =-\frac{{\left(\sum_{i}w_{i}^{2}v_{i}\right)}^{2}}{\sum_{i}w_{i}}-\sum \overset{3}{\underset{i}{w}}v_{i}^{2}\;\;\text{by the product rule.} \end{array}$$

Now, we go back and calculate the second differentials of the first two (non‐constant) terms:

$$\begin{array}{ccc} \frac{1}{2}\frac{\partial^{2}}{\partial \sigma_{1}^{2}}\sum_{i}\log(w_{i}) & =\frac{1}{2}\sum_{i}w_{i}^{2}, & \\ -\frac{1}{2}\frac{\partial^{2}}{\partial \sigma_{1}^{2}}\log\sum_{i}w_{i} & =\frac{1}{2}{\left[\frac{\sum_{i}w_{i}^{2}}{\sum_{i}w_{i}}\right]}^{2}-\frac{\sum_{i}w_{i}^{3}}{\sum_{i}w_{i}}. \end{array}$$

Hence, the expression for observed information is

$$I_{O}(\sigma_{1})=-\frac{\partial^{2}}{\partial \sigma_{1}^{2}}\log L(\sigma_{1})=\frac{{\left[\sum_{i}w_{i}^{2}(\beta_{i}-\hat{\beta })\right]}^{2}}{\sum_{i}w_{i}}+\sum w_{i}^{3}{\left(\beta_{i}-\hat{\beta }\right)}^{2}-I_{E}(\tau^{2}).$$

Finally,

$$\hat{\mathrm{Var}}(\hat{\beta })={\hat{\Phi }}_{A}=\frac{1}{\sum_{i}w_{i}}\left\{1+\frac{2\left(\frac{\sum_{i}w_{i}^{3}}{\sum_{i}w_{i}}-{\left[\frac{\sum_{i}w_{i}^{2}}{\sum_{i}w_{i}}\right]}^{2}\right)}{I(\sigma_{1})}\right\},$$

where $I(\sigma_{1})$ may be either $I_{E}(\sigma_{1})$ or $I_{O}(\sigma_{1})$.

Kenward and Roger consider inferences of the form ***L*** ***β*** for ***L*** a fixed (*l*×*p*) matrix, where *l* is the number of simultaneous inferences and *p* is the number of elements of ***β***. Here, *l*=*p*=1(since we simply wish to estimate *β*), hence ***L***=**1** also. Furthermore, since *l*=1, the test distribution is *t* rather than *F*, and the scaling factor *λ* should also be equal to 1.

To calculate approximate degrees of freedom *m*, working through the formulae of Kenward and Roger^19^(p987):

$$\begin{array}{ccc} A_{1} & =\sum_{i}\sum_{j}W_{ij}\text{tr}\left(\Theta \Phi P_{i}\Phi \right)\text{tr}\left(\Theta \Phi P_{j}\Phi \right), & \\ A_{2} & =\sum_{i}\sum_{j}W_{ij}\text{tr}\left(\Theta \Phi P_{i}\Phi \Theta \Phi P_{j}\Phi \right),\\ \Theta & =L{\left(L^{⊤}\Phi L\right)}^{-1}L^{⊤}=\Phi^{-1},\\ \Rightarrow A_{1}=A_{2} & =W_{11}P_{1}^{2}{\hat{\Phi }}_{A}^{2}\\ g & =\frac{(l+1)A_{1}-(l+4)A_{2}}{(l+2)A_{2}}=\frac{2A_{1}-5A_{1}}{3A_{1}}=-1,\\ \Rightarrow 3l+2\left(1-g\right)=7\Rightarrow c_{1} & =\frac{g}{3l+2\left(1-g\right)}=-\frac{1}{7},\\ c_{2} & =\frac{l-g}{3l+2\left(1-g\right)}=\frac{2}{7},\\ \text{and}\;\;c_{3} & =\frac{l+2-g}{3l+2\left(1-g\right)}=\frac{4}{7}. \end{array}$$

Next,

$$\begin{array}{ccc} E[F] & ={\left(1-\frac{A_{2}}{l}\right)}^{-1}={\left(1-A_{1}\right)}^{-1}, & \\ B & =\frac{1}{2l}\left(A_{1}+6A_{2}\right)=\frac{7}{2}A_{1}, \end{array}$$

and

$$\begin{array}{ccc} V[F] & =\frac{2}{l}\left\{\frac{1+c_{1}B}{{\left(1-c_{2}B\right)}^{2}\left(1-c_{3}B\right)}\right\}=\frac{2-A_{1}}{{\left(1-A_{1}\right)}^{2}\left(1-2A_{1}\right)} & \\ \Rightarrow \;\rho & =\frac{V[F]}{2E{\left[F\right]}^{2}}=\frac{1-\frac{1}{2}A_{1}}{1-2A_{1}},\\ \Rightarrow \;m & =4+\frac{l+2}{l\rho -1}=\frac{2}{A_{1}}=\frac{2}{W_{11}P_{1}^{2}{\hat{\Phi }}_{A}^{2}},\\ \text{and}\;\;\lambda & =\frac{m}{E[F]\left(m-2\right)}=1. \end{array}$$

If *W*_11_ is based on the observed information matrix, the degrees of freedom are

$$m_{O}=\frac{2\frac{{\left[\sum_{i}w_{i}^{2}(\beta_{i}-\hat{\beta })\right]}^{2}}{\sum_{i}w_{i}}+2\sum_{i}w_{i}^{3}{\left(\beta_{i}-\hat{\beta }\right)}^{2}-\sum_{i}w_{i}^{2}+2\frac{\sum_{i}w_{i}^{3}}{\sum_{i}w_{i}}-{\left[\frac{\sum_{i}w_{i}^{2}}{\sum_{i}w_{i}}\right]}^{2}}{{\hat{\Phi }}_{A}^{2}{\left(\sum_{i}w_{i}^{2}\right)}^{2}}$$

or if based on the expected information matrix:

$$m_{E}=\frac{\sum_{i}w_{i}^{2}-2\frac{\sum_{i}w_{i}^{3}}{\sum_{i}w_{i}}+{\left[\frac{\sum_{i}w_{i}^{2}}{\sum_{i}w_{i}}\right]}^{2}}{{\hat{\Phi }}_{A}^{2}{\left(\sum_{i}w_{i}^{2}\right)}^{2}}.$$
